# Primary care and COVID-19

**DOI:** 10.1017/S1463423622000196

**Published:** 2022-05-10

**Authors:** Mehmet Akman, Daksha Trivedi

**Affiliations:** 1 Professor of Family Medicine, Department of Family Medicine, Marmara University, School of Medicine, Istanbul, Turkey; 2 Reader in Applied Health Research, Centre for Research in Public Health and Community Care, University of Hertfordshire, UK

COVID-19 has had a significant impact on health care worldwide, and primary care service provision was no exception to that. There were two big challenges for primary health care (PHC): firstly, clear definition of its role to provide an effective contribution to prevention, diagnosis, and treatment of COVID-19 and re-organize accordingly; secondly, identifying novel ways of health care service provision for the non-COVID-19 patients seeking help.

In mid-2021, Kumpunen *et al.* published a paper that explore the best ways to deal with these challenges. They extracted all PHC-relevant data from the European COVID-19 Health System Response Monitor and iteratively developed an analysis framework examining the models of PHC delivery employed by PHC providers in response to the pandemic. Despite the heterogenous PHC structures and capacities across European countries, the authors identified three prevalent models of PHC delivery employed: (1) multidisciplinary primary care teams coordinating with public health to deliver the emergency response and essential services; (2) PHC providers define and identify vulnerable populations for medical and social outreach; and (3) PHC providers employ digital solutions for remote triage, consultation, monitoring, and prescriptions to avoid unnecessary contact. These results underline the importance of collaborative teamwork, digital solutions, identification of vulnerable groups, and improving access to address health inequalities magnified by the pandemic. The authors concluded with a declaration of an urgent need to capture learning from the pandemic that is specific to PHC services and to turn these into strategic action plans to strengthen preparedness for future outbreaks and better respond to the contemporary health challenges (Kumpunen *et al.*, 2021). We hope that this collection goes some way to providing such learning to support the development of pandemic-ready PHC services.

The call for this Special Issue on the primary care response to COVID-19 was launched approximately 1 year after the outbreak of the pandemic and now we are very happy to share the whole collection approximately 2 years after the outbreak. In other words, the papers included reflecting the more organized efforts of the fight against COVID-19 rather than initial, possibly premature, reactions. Therefore, articles in this collection can guide researchers, service providers, and policymakers in the development of strategies for better preparedness for future pandemics. We had a remarkable number of submissions for the call from all over the world, 20 of them are accepted to date. Among these, we have 1 position paper from WONCA Europe on telemedicine, 2 scoping reviews, 2 development papers, 3 short reports, and 12 research papers. Country of origin variability is very high among these papers, and they are from four different continents. We would like to thank all authors for their outstanding contributions. This research collection sets the benchmark for developing integrated models of care including for recovery from COVID-19. It opens up the debate around supporting primary care in the growing demand for post-COVID-19 care, particularly around accessing appropriate care (NHS England, 2021) so that we provide the best service for those we care for.

In general, three major categories/themes emerged from the analysis of the accepted papers: impact of the pandemic on primary care service provision; digital solutions during a pandemic; and primary care response to COVID-19. The latter is the main focus of the collection with 10 articles in this category. Among the papers related to primary care response, Kinder *et al.* (2021) tackle one of the most burning issues on integration of public health and primary care for a stronger response to future pandemics. These authors surveyed more than a thousand primary care respondents from over a hundred countries, and they concluded that weak/no integration of public health and primary care is a contributing factor to the bypass of the primary care clinics as the first contact of care during pandemics. Hence, they suggest strengthening the surveillance function of primary care. Another interesting paper under this category is a development paper by Johansen *et al.* (2021). They explored an operational tool for policymakers developed by WHO, and its pilot results are very promising for identifying the critical challenges that need to be addressed for an efficient response to COVID-19.

Finally, we would like to thank the authors of the other three editorials, Professor Jan De Maeseneer, Professor Felicity Goodyear-Smith, and the EFPC author team for accepting our invitation and contributing to this Special Issue. Enjoy reading!
